# Role of Reduced Sarco-Endoplasmic Reticulum Ca^2+^-ATPase Function on Sarcoplasmic Reticulum Ca^2+^ Alternans in the Intact Rabbit Heart

**DOI:** 10.3389/fphys.2021.656516

**Published:** 2021-05-11

**Authors:** Lianguo Wang, Rachel C. Myles, I-Ju Lee, Donald M. Bers, Crystal M. Ripplinger

**Affiliations:** ^1^Department of Pharmacology, School of Medicine, University of California, Davis, Davis, CA, United States; ^2^Institute of Cardiovascular and Medical Sciences, University of Glasgow, Glasgow, United Kingdom

**Keywords:** sarco-endoplasmic reticulum Ca^2+^-ATPase, sarcoplasmic reticulum Ca^2+^, optical mapping, alternans, arrhythmia

## Abstract

Sarcoplasmic reticulum (SR) Ca^2+^ cycling is tightly regulated by ryanodine receptor (RyR) Ca^2+^ release and sarco-endoplasmic reticulum Ca^2+^-ATPase (SERCA) Ca^2+^ uptake during each excitation–contraction coupling cycle. We previously showed that RyR refractoriness plays a key role in the onset of SR Ca^2+^ alternans in the intact rabbit heart, which contributes to arrhythmogenic action potential duration (APD) alternans. Recent studies have also implicated impaired SERCA function, a key feature of heart failure, in cardiac alternans and arrhythmias. However, the relationship between reduced SERCA function and SR Ca^2+^ alternans is not well understood. Simultaneous optical mapping of transmembrane potential (V_m_) and SR Ca^2+^ was performed in isolated rabbit hearts (*n* = 10) using the voltage-sensitive dye RH237 and the low-affinity Ca^2+^ indicator Fluo-5N-AM. Alternans was induced by rapid ventricular pacing. SERCA was inhibited with cyclopiazonic acid (CPA; 1–10 μM). SERCA inhibition (1, 5, and 10 μM of CPA) resulted in dose-dependent slowing of SR Ca^2+^ reuptake, with the time constant (*tau*) increasing from 70.8 ± 3.5 ms at baseline to 85.5 ± 6.6, 129.9 ± 20.7, and 271.3 ± 37.6 ms, respectively (*p* < 0.05 vs. baseline for all doses). At fast pacing frequencies, CPA significantly increased the magnitude of SR Ca^2+^ and APD alternans, most strongly at 10 μM (pacing cycle length = 220 ms: SR Ca^2+^ alternans magnitude: 57.1 ± 4.7 vs. 13.4 ± 8.9 AU; APD alternans magnitude 3.8 ± 1.9 vs. 0.2 ± 0.19 AU; *p* < 0.05 10 μM of CPA vs. baseline for both). SERCA inhibition also promoted the emergence of spatially discordant alternans. Notably, at all CPA doses, alternation of SR Ca^2+^ release occurred prior to alternation of diastolic SR Ca^2+^ load as pacing frequency increased. Simultaneous optical mapping of SR Ca^2+^ and V_m_ in the intact rabbit heart revealed that SERCA inhibition exacerbates pacing-induced SR Ca^2+^ and APD alternans magnitude, particularly at fast pacing frequencies. Importantly, SR Ca^2+^ release alternans always occurred before the onset of SR Ca^2+^ load alternans. These findings suggest that even in settings of diminished SERCA function, relative refractoriness of RyR Ca^2+^ release governs the onset of intracellular Ca^2+^ alternans.

## Introduction

In mammalian hearts, Ca^2+^ release from and reuptake into the sarcoplasmic reticulum (SR) is tightly regulated for proper excitation–contraction coupling (ECC) (Bers, 2002a). At steady state, during each ECC cycle, Ca^2+^ is released from the SR through ryanodine receptors (RyRs) during systole, and the same amount of Ca^2+^ is taken back up by the sarco-endoplasmic reticulum Ca^2+^-ATPase (SERCA) pump during diastole. At rapid rates, beat-to-beat alternation in the amount of Ca^2+^ released from the SR has been demonstrated to underlie the development of repolarization alternans (Chudin et al., 1999; Diaz et al., 2004; Goldhaber et al., 2005; Picht et al., 2006; Laurita and Rosenbaum, 2008; Bayer et al., 2010; Alvarez-Lacalle et al., 2013), which can lead to lethal ventricular arrhythmias in patients (Gehi et al., 2005; Verrier et al., 2011). Several recent studies have shown that RyR function and expression play a key role in the onset of Ca^2+^ alternans (Kornyeyev et al., 2012; Wang et al., 2014; Zhong et al., 2016; Sun et al., 2018; Zhong et al., 2018). For example, using optical mapping of free intra-SR Ca^2+^ in the intact rabbit heart, we showed that as heart rate increases, SR Ca^2+^ release begins to alternate without appreciable changes in diastolic SR Ca^2+^ load, suggesting that refractoriness of RyR Ca^2+^ release governs the onset of alternans (Wang et al., 2014). Indeed, in that study, sensitizing RyR with low-dose caffeine delayed the onset and reduced the magnitude of SR Ca^2+^ and resulting action potential (AP) duration (APD) alternans (Wang et al., 2014). Several recent studies in mouse hearts and myocytes have revealed that either reduced expression or loss of function of RyR exacerbates intracellular Ca^2+^ alternans (Zhong et al., 2016; Sun et al., 2018), whereas gain of function in RyR reduces Ca^2+^ alternans (Kornyeyev et al., 2012; Sun et al., 2018). However, the relationship between reduced SERCA function, as occurs in failing hearts, and SR Ca^2+^ alternans is not as straightforward.

Impaired SERCA function occurs in heart failure (HF) (Sakata et al., 2007; Kawase et al., 2008; Jessup et al., 2011; Zsebo et al., 2014) and is known to potentiate intracellular Ca^2+^ alternans, presumably due to insufficient Ca^2+^ reuptake during diastole and subsequent alternation of SR Ca^2+^ load (Wilson et al., 2009). Because SR Ca^2+^ release is steeply dependent on SR Ca^2+^ load (Bers, 2001, 2002a, 2014), insufficient Ca^2+^ reuptake and beat-to-beat alternation in SR Ca^2+^ load could therefore cause alternation of SR Ca^2+^ release (Diaz et al., 2004; Xie et al., 2008; Zhou et al., 2016). Indeed, detailed mechanistic studies in isolated cardiomyocytes have confirmed this mechanism (Diaz et al., 2004). Studies in failing hearts have also shown that decreased SERCA expression or activity is associated with Ca^2+^ alternans, and overexpression of the cardiac SERCA pump can suppress Ca^2+^ alternans (Wan et al., 2005; Cutler et al., 2009, 2012). On the other hand, mathematical simulations suggest that severely reducing the activity of SERCA may actually suppress rather than promote Ca^2+^ alternans (Weiss et al., 2006; Qu et al., 2013, 2016). Indeed, a recent experimental study confirmed that severe pharmacological inhibition of SERCA suppressed alternans, but promoting SERCA function via phospholamban knock-out had only a minor effect on Ca^2+^ alternans in both wild-type and RyR loss-of-function mice (Sun et al., 2018). A recent study in the rat heart shows that atrial SERCA overexpression or inhibition had no effect on cardiac alternans (Nassal et al., 2015). These conflicting observations suggest that the effect of altered SERCA activity on the genesis of SR Ca^2+^ alternans is complex and warrants further investigation.

To address the role of reduced SERCA function in contributing to SR Ca^2+^ alternans and subsequent arrhythmogenic APD alternans, we performed optical mapping of free intra-SR Ca^2+^ concomitantly with transmembrane potential (V_m_) in intact rabbit hearts. We assessed the impact of reduced SERCA function using cyclopiazonic acid (CPA), a SERCA inhibitor (Szentesi et al., 2004), on SR Ca^2+^ alternans and evaluated whether alternans were driven by beat-to-beat changes in SR Ca^2+^ release and/or diastolic SR Ca^2+^ load. We also determined the relationship between pacing frequency and degree of SERCA inhibition on resulting SR Ca^2+^ and APD alternans magnitude.

## Materials and Methods

### Ethical Approval

All procedures involving animals were approved by the Animal Care and Use Committee of the University of California, Davis (Reference No. 20991), and adhered to the Guide for the Care and Use of Laboratory Animals published by the National Institutes of Health (NIH Publication N0. 85-23, revised 2011). Male New Zealand White rabbits (3.0–3.5 kg, *n* = 10; Charles River Laboratories) were housed on a 12 h light–dark cycle and given access to food and water *ad libitum.*

### Whole-Heart Langendorff Perfusion

Rabbit hearts were Langendorff-perfused as described previously (Wang et al., 2014, 2015; Murphy et al., 2017). Briefly, rabbits were administered heparin (1,000 units IV) and were anesthetized with pentobarbital sodium (50 mg/kg IV). After deep anesthesia was achieved, evidenced by lack of eye-blink and foot withdrawal reflexes, a median sternotomy was performed with a vertical midline incision from the substernal notch to the xiphoid process. Hearts were rapidly removed and placed in 200 ml of ice-cold cardioplegia solution (composition in mmol/L: NaCl 110, CaCl_2_ 1.2, KCl 16, MgCl_2_ 16, and NaHCO_3_ 10). Following cannulation of the aorta, Langendorff perfusion was initiated with oxygenated (95% O_2_, 5% CO_2_) Tyrode’s solution of the following composition (in mmol/L): NaCl 128.2, CaCl_2_ 1.3, KCl 4.7, MgCl_2_ 1.05, NaH_2_PO_4_ 1.19, NaHCO_3_ 20, and glucose 11.1 (pH 7.4 ± 0.05). The perfusate was pumped from a reservoir with 2 L of Tyrode’s solution through an in-line filter and two bubble traps before passing via the cannula to the heart and then recirculated from the perfusion chamber back to the reservoir and re-gassed. Flow rate (∼30 ml/min) was adjusted to maintain a perfusion pressure of 60–70 mmHg. The heart was securely positioned supine in the perfusion chamber. The perfusion apparatus was temperature controlled with heated baths used for the perfusate and a water-jacketed perfusion chamber. Two Ag/AgCl disc electrodes were positioned in the bath to record an electrocardiogram (ECG) analogous to a lead I configuration. A bipolar pacing electrode was positioned on the base of the left ventricular (LV) epicardium.

### Dual Optical Mapping of SR Ca^2 +^ and V_m_

Optical mapping of intra-SR free [Ca^2+^] and transmembrane potential (V_m_) was performed as previously described in detail (Wang et al., 2014, 2015; Murphy et al., 2017; Wang and Ripplinger, 2019). After a 10-min equilibration period, blebbistatin (Tocris Bioscience, Ellisville, MO; 10–20 μM) was added to the perfusate to reduce energy demands of the heart during dye loading (Wengrowski et al., 2013) and to eliminate motion artifact during optical recordings (Fedorov et al., 2007). Hearts were then switched to a recirculating perfusate (200 ml) and loaded by retrograde perfusion with Tyrode’s solution containing Fluo-5N, acetoxymethyl ester (Fluo-5N AM; 5 μM, dissolved in dimethyl sulfoxide and Pluronic F-127, 20% wt/vol, Invitrogen; Carlsbad, CA, United States) for 60 min at room temperature (Kornyeyev et al., 2010), followed by 15 min washout at 37°C to remove residual Fluo-5N AM. Hearts were subsequently stained with the voltage-sensitive dye RH237 [Invitrogen, Carlsbad, CA, United States; 50 μl of 1 mg/ml in dimethyl sulfoxide (DMSO)]. All experiments were performed at 37°C.

The anterior epicardial surface was excited using LED light sources centered at 470 nm (Mightex, Pleasanton, CA, United States) and bandpass filtered from 475 to 495 nm (Semrock, Rochester, NY, United States). The emitted fluorescence was collected through a THT-macroscope (SciMedia) and split with a dichroic mirror at 545 nm (Omega, Brattleboro, VT, United States). The longer wavelength moiety, containing the V_m_ signal, was longpass filtered at 700 nm; and the shorter wavelength moiety, containing the SR Ca^2+^ signal, was bandpass filtered with a 32-nm filter centered at 518 nm (Omega, Brattleboro, VT, United States). The emitted fluorescence signals were recorded using two CMOS cameras (MiCam Ultima-L, SciMedia, Costa Mesa, CA, United States) with a sampling rate of 0.5–1 kHz and 100 × 100 pixels with a field of view of 31 × 31 mm.

### Experimental Protocol

Baseline electrophysiological parameters were determined during LV epicardial pacing at a pacing cycle length (PCL) of 300 ms using a 2-ms pulse at twice the diastolic threshold. To induce alternans, the PCL was decremented in 20-ms steps. The effects of pharmacological SERCA inhibition were determined by adding CPA (1–10 μM) to the perfusate. Relative refractoriness of RyR Ca^2+^ release was measured as recovery of SR Ca^2+^ release with a single extra-stimulus (S1–S2 pacing protocol).

### Data Analysis and Statistics

Data analysis was performed using two commercially available analysis programs (*BV_Analyze*, Brainvision, Tokyo, Japan; and *Optiq*, Cairn, United Kingdom) and a free optical mapping analysis software ElectroMap (O’Shea et al., 2019). V_m_ and SR Ca^2+^ datasets were spatially aligned (using heart images from each camera obtained during white light illumination) and processed with a Gaussian spatial filter (radius 3 pixels). For both APs and SR Ca^2+^ transients, activation time was determined at 50% of the maximal amplitude (or time at 50% of nadir for SR Ca^2+^). For APs, repolarization time at 80% return to baseline was used to calculate APD (APD_8__0_). SERCA function was quantified using the time constant (τ) of a single exponential fit to the recovery portion of the SR Ca^2+^ trace (from 5 to 90% recovery).

The spectral method, which has been used clinically for detecting micro-volt T-wave alternans (Verrier et al., 2011), was used to detect the presence of significant APD and SR Ca^2+^ alternans as previously described (Myles et al., 2011). The spectral method was chosen due to its high sensitivity and relative immunity to noise. Briefly, at each pacing frequency, APs or SR Ca^2+^ transients from a ∼4-s recording were aligned in time using the pacing artifact as a reference point, resulting in a two-dimensional matrix of signals, *AP*(*n*,*t*), where *n* is the beat number and *t* is time. A fast Fourier transform (FFT) was used to compute the power spectra across beats for each *t*, and the spectra were summed. The alternans magnitude was then defined as the resulting amplitude of the summed spectra at 0.5 cycles/beat. This approach allowed us to determine if an area within the mapping field of view was experiencing significant APD or SR Ca^2+^ alternans (greater than the background noise levels) as well as the spatial extent of significant alternans. A magnitude of ≥ 2 was used as the minimum threshold for significant APD or SR Ca^2+^ alternans, corresponding to a beat-to-beat change in APD_90_ ≥ 5 ms or beat-to-beat change in SR Ca^2+^ release amplitude ≥ 5%, respectively.

To more precisely differentiate between the onset of diastolic SR Ca^2+^ load alternans and SR Ca^2+^ release alternans, quantification of the SR Ca^2+^ transient was also performed, as previously described (Wang et al., 2014). The amplitude of SR Ca^2+^ release alternans was calculated as 1 minus the ratio of the average small beat (S) release amplitude to the average large beat (L) release amplitude (1 - S/L) during a 1- to 2-s recording. The amplitude of diastolic SR Ca^2+^ load alternans was calculated as the average difference between diastolic levels (D) of S and L beats divided by the average L amplitude (D/L) during a 1- to 2-s recording. Data are expressed as mean ± standard deviation (SD) and were compared using a one-way ANOVA with Bonferroni *post-hoc* test for multiple groups or a Student *t*-test when only two groups were compared. *p* < 0.05 was considered statistically significant.

## Results

### Effects of Sarco-Endoplasmic Reticulum Ca^2+^-ATPase Inhibition on V_m_ and Sarcoplasmic Reticulum Ca^2+^

**FIGURE 1:**
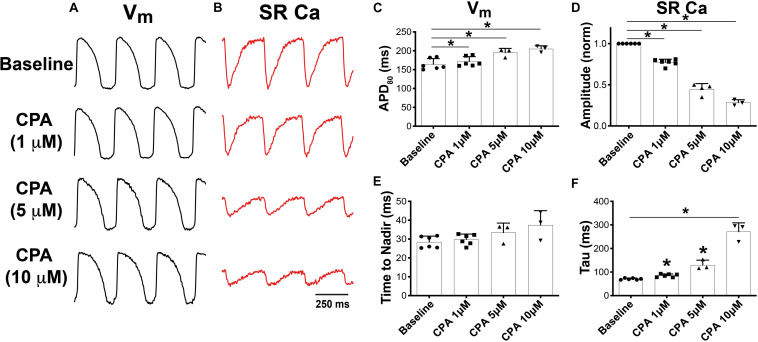
Dual optical mapping of V_m_ **(A)** and sarcoplasmic reticulum (SR) Ca^2+^ **(B)** during sarco-endoplasmic reticulum Ca^2+^-ATPase (SERCA) inhibition with cyclopiazonic acid (CPA) and pacing at a pacing cycle length (PCL) of 300 ms. CPA caused a dose-dependent increase in APD_80_ **(C)** and decrease in normalized SR Ca^2+^ transient amplitude **(D)**. CPA tended to prolong the time to nadir of SR Ca^2+^ release **(E)** and significantly prolonged SR Ca^2+^ reuptake time (*tau*, **F**). Mean ± SD. *N* = 3–6 (*p < 0.05 vs. baseline).

To determine the role of SERCA function on V_m_ and SR Ca^2+^, increasing doses of CPA, a specific SERCA inhibitor (Seidler et al., 1989; Szentesi et al., 2004), were added to the perfusate, while simultaneous imaging of RH237 (V_m_) and Fluo-5N (SR Ca^2+^) was performed at 300-ms PCL (Figures 1A,B). As expected, CPA (1, 5, and 10 μM) prolonged APD (Figure 1C) and decreased the relative amplitude of the normalized SR Ca^2+^ transient in a dose-dependent manner (Figure 1D). These findings are consistent with previous observations in adult guinea pig ventricular myocytes in the presence of CPA (Szentesi et al., 2004). Furthermore, CPA tended to increase the SR Ca^2+^ time to nadir (indicative of SR Ca^2+^ release, Figure 1E) and caused a dose-dependent increase in SR Ca^2+^ reuptake time (*tau*, Figure 1F), with *tau* increasing from 70.8 ± 3.5 ms at baseline to 85.5 ± 6.6, 129.9 ± 20.7, and 271.3 ± 37.6 ms (*p* < 0.05 vs. baseline for all doses). CPA did not impact mean conduction velocity (CV: 54.9 ± 1.6 cm/s at baseline vs. 54.8 ± 4.6, 54.7 ± 3.2, and 53.8 ± 2.8 cm/s for 1, 5, and 10 μM, *p* = NS). The marked prolongation of APD in the presence of CPA suggests that alterations in SR Ca^2+^ reuptake feed back onto V_m_ to influence AP dynamics, potentially due to a reduction in Ca^2+^-dependent inactivation of L-type Ca^2+^ current (I_CaL_) and consequent increased I_CaL_ during the AP plateau.

### Sarco-Endoplasmic Reticulum Ca^2+^-ATPase Inhibition Worsens Pacing-Induced Action Potential Duration and Sarcoplasmic Reticulum Ca^2+^ Alternans

**FIGURE 2:**
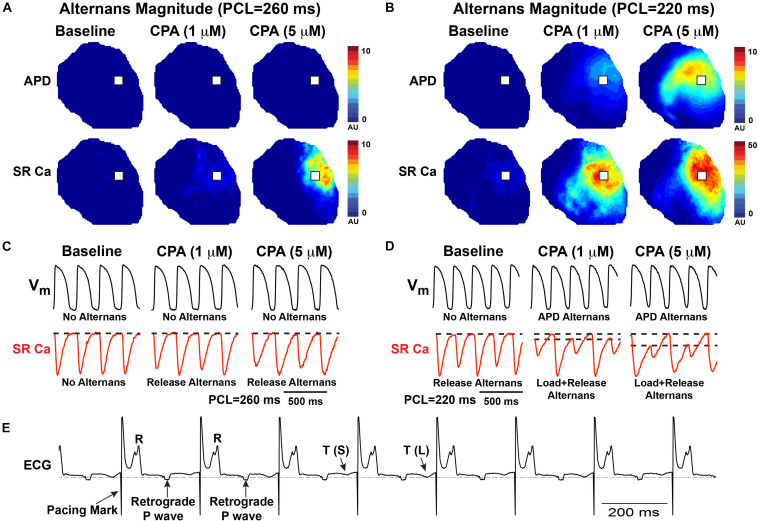
Both action potential duration (APD) and sarcoplasmic reticulum (SR) Ca^2+^ alternans progressively worsen with increased pacing frequency as shown by the alternans magnitude maps **(A,B)** and in example optical traces **(C,D)** from the location indicated with a white box in **(A,B)**. In this example, cyclopiazonic acid (CPA) (1–5 μM) caused a dose-dependent increase in the magnitude of SR Ca^2+^ and APD alternans and an increase in the pacing cycle length (PCL) at which alternans occurred. **(E)** Example ECG showing T-wave alternans—small (S) vs. large (L) T-wave.

**FIGURE 3:**
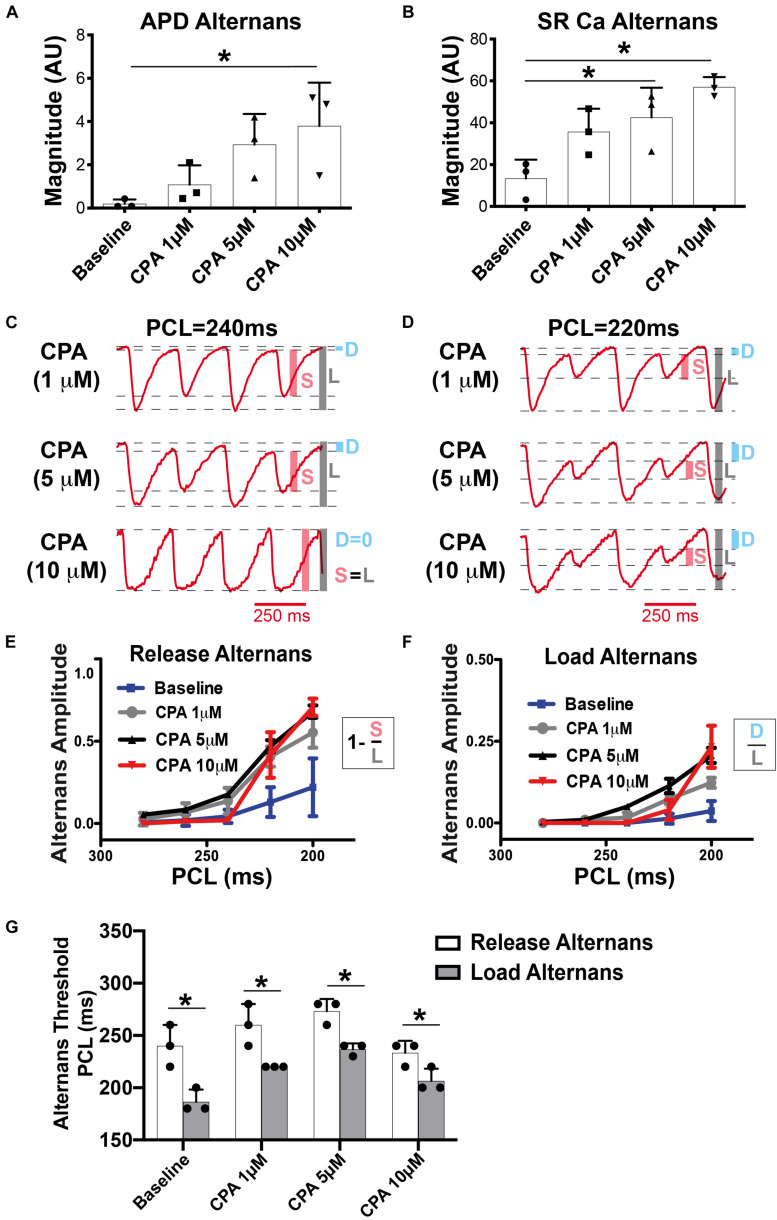
At pacing cycle length (PCL) = 220 ms, cyclopiazonic acid (CPA) increased the magnitude of both action potential duration (APD) **(A)** and sarcoplasmic reticulum (SR) Ca^2+^ **(B)** alternans. Mean ± SD. *N* = 3 (*p < 0.05 vs. Baseline). **(C,D)** Example SR Ca^2+^ traces at a slower **(C)** and faster **(D)** PCL. High-dose CPA (10 μM) tended to decrease alternans magnitude at slower rates, while increasing alternans magnitude at faster rates. **(E,F)** Quantification of release **(E)** and load **(F)** alternans according to the equations indicated. Shaded bars in **(C,D)** show approximate measurements for calculations in **(E,F)**. S, small; L, large; D, diastolic level. **(G)** Under all conditions, release alternans emerged at a longer PCL than load alternans. Mean ± SD. *N* = 3 (*p < 0.05 vs. corresponding threshold for release alternans).

To induce alternans, hearts were paced by decrementing the PCL. Consistent with previous reports, SERCA inhibition (1 and 5 μM of CPA) increased the incidence and magnitude of both APD and SR Ca^2+^ alternans (Wan et al., 2005; Nivala et al., 2015; Weinberg, 2016). Example maps of the alternans magnitude at two PCLs are shown in Figures 2A,B, along with corresponding optical V_m_ and SR Ca^2+^ traces (Figures 2C,D). Consistent with our previous studies, significant SR Ca^2+^ alternans emerged prior to the onset of APD alternans (i.e., at longer PCLs) (Wang et al., 2014). In the example shown, at baseline, SR Ca^2+^ alternans was induced at PCL = 220 ms without significant APD alternans (Figures 2B,D, top vs. bottom left traces). CPA at 1 and 5 μM dose-dependently shifted the alternans threshold to a longer PCL and increased the magnitude of both APD and SR Ca^2+^ alternans. Indeed, when the alternans magnitude was quantified at PCL = 220 ms, the magnitude of both APD and SR Ca^2+^ alternans tended to increase in a dose-dependent manner (Figures 3A,B). However, detailed analysis of SR Ca^2+^ kinetics and the frequency dependence of alternans revealed more complex behaviors.

### Effects of Sarco-Endoplasmic Reticulum Ca^2+^-ATPase Inhibition on Sarcoplasmic Reticulum Ca^2+^ Load and Release Alternans

Although spectral analysis allows for sensitive detection of both APD and SR Ca^2+^ alternans, it does not differentiate SR Ca^2+^ diastolic load from release alternans. To more precisely investigate the role of SERCA inhibition, detailed quantification of the SR Ca^2+^ transient was performed as shown in Figures 3C–F. We have previously shown in the normal heart that SR Ca^2+^ release alternans always occur at slower PCLs, prior to the onset of SR Ca^2+^ load alternans, indicating the prominent role of RyR refractoriness in governing the onset of alternans (Wang et al., 2014). Our working hypothesis was that SERCA inhibition with CPA would lead to insufficient SR Ca^2+^ reuptake during diastole, causing alternation of diastolic SR Ca^2+^ load, and that load and release alternans may therefore emerge simultaneously. Contrary to this hypothesis, at all doses of CPA, alternation of SR Ca^2+^ release occurred prior to the onset of any detectable alternation in SR Ca^2+^ load (Figures 2C,D, 3E–G). Interestingly, high-dose CPA (10 μM) tended to suppress both load and release alternans at slower pacing frequencies (Figures 3C,E–G) but caused larger magnitude load and release alternans at faster pacing frequencies (similar to 5 μM of CPA, Figures 3E,F). Yet even with high-dose CPA, significant SR Ca^2+^ release alternans occurred before the onset of detectable SR Ca^2+^ load alternans (Figure 3G), suggesting that RyR refractoriness plays a key role in the genesis of cardiac alternans, even when SERCA function is significantly reduced. Indeed, at the lower [Ca^2+^]_SR_ associated with CPA-dependent SERCA inhibition, the RyR refractory period might be prolonged, opposite to the shortening of refractoriness at very high [Ca^2+^]_SR_ levels (Bers, 2002b).

### Sarco-Endoplasmic Reticulum Ca^2+^-ATPase Inhibition Increases Relative Ryanodine Receptor Refractoriness

**FIGURE 4:**
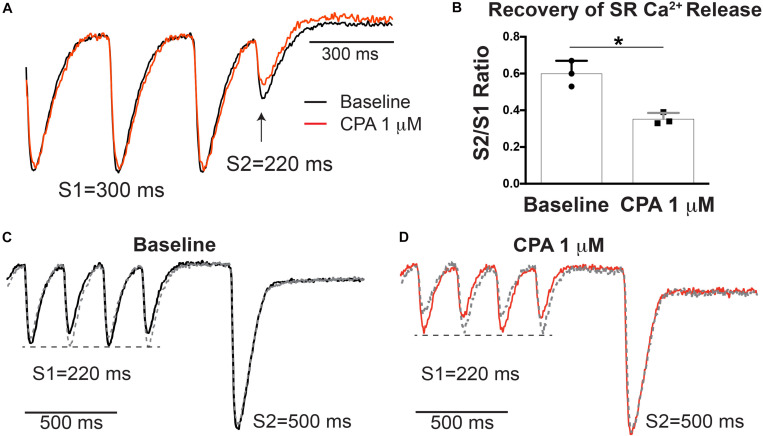
Sarco-endoplasmic reticulum Ca^2+^-ATPase (SERCA) inhibition with cyclopiazonic acid (CPA) increased relative ryanodine receptor (RyR) refractoriness **(A)**. **(B)** S2/S1 ratio (S1 = 300, S2 = 220 ms) was significantly decreased with 1 μM of CPA. Mean ± SD. *N* = 3 (*p < 0.05 vs. baseline). **(C,D)** Alternative S1–S2 protocol in which alternans was induced at S1 = 220 ms and a longer S2 (500 ms) was applied. The pacing protocol was repeated so that the last S1 was either a small (solid) or large (dashed) release. A similarly large sarcoplasmic reticulum (SR) Ca^2+^ release occurred after a large or small release at baseline **(C)** and following 1 μM of CPA **(D)**, indicating similar SR Ca^2+^ load after small and large beats.

To determine if SERCA inhibition modifies relative RyR refractoriness and recovery of SR Ca^2+^ release, an S1–S2 pacing protocol was performed with low-dose (1 μM) CPA (Figure 4A). At S1 = 300 ms, there was no SR Ca^2+^ alternans at baseline or with low-dose CPA, but there is incomplete recovery of SR Ca^2+^ release at S2 = 220 ms in both groups (Figure 4A). Notably, 1 μM of CPA caused a stronger suppression of the S2-induced SR Ca^2+^ release when normalized to the S1 release (S2/S1 ratio, Figure 4B), consistent with a prolongation in relative RyR refractoriness upon SERCA inhibition. An alternative S1–S2 protocol was also performed, where the S1 train at a shorter PCL (220 ms) induces SR Ca^2+^ alternans, followed by a long S2 pause to ensure more complete RyR recovery (Figures 4C,D). This protocol was repeated so that the longer S2 interval occurred after both large and small SR Ca^2+^ release. In response to the long S2 interval, a similar amplitude SR Ca^2+^ release occurred, regardless of the amplitude of SR Ca^2+^ release at the last S1 beat (large or small), indicating that SR Ca^2+^ content does not appreciably alternate from beat to beat and that at this pacing frequency, availability of RyR for Ca^2+^ release is a key contributor to alternans both with and without CPA.

### Sarco-Endoplasmic Reticulum Ca^2+^-ATPase Inhibition Promotes Spatially Discordant Alternans

**FIGURE 5:**
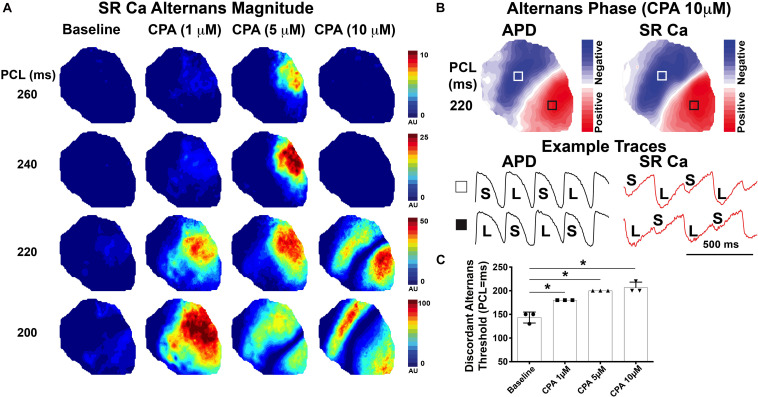
Inhibition of sarco-endoplasmic reticulum Ca^2+^-ATPase (SERCA) with cyclopiazonic acid (CPA) promoted the emergence of spatially discordant alternans at slower pacing cycle lengths (PCL). **(A)** Magnitude of sarcoplasmic reticulum (SR) Ca^2+^ alternans at progressively faster PCLs. Spatial discordance emerges at 220-ms PCL for 10 μM of CPA and at 200-ms PCL for 5 μM CPA, while baseline and 1 μM of CPA remain spatially concordant over the PCLs shown. **(B)** Alternans phase with the nodal line indicated in white where alternans transitions from small:large to large:small. Optical V_m_ and SR Ca^2+^ traces are from the regions indicated with white and black boxes. **(C)** Pacing threshold for the induction of spatial discordance was significantly increased with CPA. Mean ± SD. *N* = 3 (*p < 0.05 vs. baseline).

Further investigation of spatial alternans dynamics shows that CPA also promoted an earlier emergence of spatially discordant alternans. Figure 5A illustrates the emergence of SR Ca^2+^ alternans with increasing doses of CPA and progressively faster PCLs. Spatial discordance occurs when a clear nodal line is observed (5 μM of CPA at 200 ms and 10 μM of CPA at 220 and 200 ms) and was confirmed by assessing alternans phase (Figure 5B). The pacing threshold for emergence of spatial discordance increased with increasing doses of CPA (Figure 5C), and in all cases, spatial discordance of both APD and SR Ca^2+^ alternans occurred at the same PCL. The fact that pacing thresholds for spatial discordance are the same for APD and SR Ca^2+^ indicates that V_m_ and SR Ca^2+^ remain in-phase with each other (long APD corresponds to larger SR Ca^2+^ transient and *vice versa*) even when the heart is spatially out-of-phase (long APD and large SR Ca^2+^ transient in one location; short APD and smaller SR Ca^2+^ transient in another location). Spatially discordant alternans are known to create very large gradients in repolarization, setting the stage for unidirectional conduction block and the induction of reentry (Pastore et al., 1999; Walker and Rosenbaum, 2005; Hayashi et al., 2007; Wilson and Rosenbaum, 2007).

## Discussion

Using dual optical mapping of V_m_ and SR Ca^2+^, we show that modest inhibition of SERCA increases relative RyR refractoriness and worsens pacing-induced SR Ca^2+^ and APD alternans. Importantly, even under conditions of SERCA inhibition, SR Ca^2+^ release alternans always occurred prior to the onset of appreciable SR Ca^2+^ load alternans or APD alternans. Severe inhibition of SERCA function with high-dose CPA tended to suppress SR Ca^2+^ alternans at slower pacing but worsened alternans at faster pacing frequencies. All doses of CPA also promoted the emergence of spatial discordance. While these data demonstrate that both SERCA and RyR function are key players, the fact that significant SR Ca^2+^ release alternans always occurs before the onset of SR Ca^2+^ load alternans suggests that encroachment on RyR refractoriness *per se* predominates as the initiator of Ca^2+^ transients alternans. Furthermore, we find that slowed SR Ca^2+^ uptake and lower SR Ca^2+^ content shift that refractoriness threshold to longer PCLs.

### Simultaneous Mapping of V_m_ and Sarcoplasmic Reticulum Ca^2+^ in the Intact Heart

We previously developed methodology using the voltage-sensitive dye, RH237, and low-affinity Ca^2+^ indicator, Fluo-5N AM, to map V_m_ and SR Ca^2+^ simultaneously in the intact heart (Wang et al., 2014, 2015; Murphy et al., 2017). Fluo-5N has a dissociation constant (K_*d*_) around 400 μM and therefore exhibits minimal fluorescence in the cytosol compared to the SR lumen, where the Ca^2+^ content is at or near the millimolar range. Thus, SR Ca^2+^ dynamics can be assessed independently from transmembrane Ca^2+^ flux. Both fluorescent indicators are excited with blue light (488 nm), and the emission wavelengths are split and recorded on two separate high-speed detectors, allowing for precise beat-to-beat mapping of V_m_ and SR Ca^2+^ dynamics at high spatial and temporal resolutions (Wang et al., 2014). As shown in Figure 1, in addition to expected prolongation of SR Ca^2+^ reuptake (*tau*), SERCA inhibition also dose-dependently prolonged APD and decreased the relative amplitude of SR Ca^2+^ release. Decreased SR Ca^2+^ transient amplitude is suggestive of decreased SR Ca^2+^ content, which would be expected with SERCA inhibition; however, Fluo-5N Ca^2+^ signals are uncalibrated, and therefore, signal amplitudes can only be used to ascertain relative differences.

### Impact of Sarco-Endoplasmic Reticulum Ca^2+^-ATPase Inhibition on V_m_ and Sarcoplasmic Reticulum Ca^2+^ Alternans

Under normal conditions and stable heart rate, diastolic SR Ca^2+^ load varies little from beat to beat, indicating a precise balance between RyR Ca^2+^ release and SERCA reuptake during each ECC cycle. We have previously shown in normal rabbit hearts that as heart rate increases, beat-to-beat alternation of SR Ca^2+^ release occurs prior to appreciable alternation of SR Ca^2+^ load and that relative refractoriness of RyR governs the onset of arrhythmogenic alternans (Wang et al., 2014). However, SERCA function has also been implicated in cardiac alternans, particularly in HF where SERCA expression or activity is reduced and alternans severity is increased (Wan et al., 2005; Cutler et al., 2009, 2012). The objective of the present study was to test the relatively straightforward hypothesis that SERCA inhibition with CPA would worsen alternans and would do so via insufficient SR Ca^2+^ reuptake during diastole, causing alternation of diastolic SR Ca^2+^ load, and that load and release alternans may therefore emerge simultaneously. While CPA did indeed worsen alternans, release alternans still preceded load alternans, causing us to reject this simple explanatory hypothesis.

One consistent and somewhat unexpected finding of the present study was that SR Ca^2+^ release *always* alternated prior to any discernable alternation in diastolic SR Ca^2+^ load under all conditions assessed (Figures 2, 3G). Therefore, even though SR Ca^2+^ reuptake was impaired and slowed with CPA, insufficient diastolic SR refilling by itself does not appear to be a primary driver of alternans onset. A mechanistic clue was that even mild SERCA inhibition slowed the relative recovery of RyR Ca^2+^ release measured with an S1–S2 protocol (Figure 4), and increased RyR refractoriness is consistent with increased alternans magnitude. The magnitude and recovery of RyR Ca^2+^ release are known to be highly dependent on SR Ca^2+^ load, and although we assume that SERCA inhibition decreases SR Ca^2+^ content, we cannot measure absolute SR Ca^2+^ concentration with our method. However, recent data indicate that the *velocity* of SR Ca^2+^ refilling may affect SR Ca^2+^ release restitution, independent of SR Ca^2+^ load (Cely-Ortiz et al., 2020), and this mechanism may also be involved here.

At lower doses (1 and 5 μM), CPA tended to increase the magnitude of SR Ca^2+^ and APD alternans, as well as increase the PCL at which alternans first emerged (Figures 2, 3). High-dose (10 μM) CPA, however, tended to reduce alternans at slow PCLs but worsened alternans at fast PCLs (Figures 3, 5). These somewhat conflicting effects raise an important question of how severely reduced SERCA function suppresses SR Ca^2+^ alternans at slower pacing (e.g., PCL = 240–260 ms) and yet promotes SR Ca^2+^ alternans at faster pacing (e.g., PCL = 200–220 ms). One possible explanation is that greatly impaired SERCA function (by ∼75% based on refilling *tau* values in Figure 1F) may reduce SR Ca^2+^ content and release dramatically (Millet et al., 2021), especially when the pacing frequency is slow, masking alternans at slower rates. However, as pacing frequency increases, SR Ca^2+^ load also increases (Wang et al., 2014), which in turn would increase the magnitude of SR Ca^2+^ release and make alternans more apparent. Alternatively, rapid pacing may cause diastolic cytosolic Ca^2+^ elevation, which in turn triggers Ca^2+^-calmodulin-dependent inactivation of RyR and, consequently, an imbalance of SR Ca^2+^ release and reuptake (Wei et al., 2020). Thus, the role of SERCA activity in either suppressing or promoting SR Ca^2+^ alternans may depend on relative changes in SR Ca^2+^ content at various pacing frequencies.

Moreover, the overall magnitude of *intracellular* Ca^2+^ alternans will also depend on transmembrane Ca^2+^ flux (i.e., I*_*Ca*_*_L_) and intracellular Ca^2+^ concentration, which were not assessed here but are also dependent on SERCA and RyR activity. Indeed, a previous study in the isolated rabbit working heart showed that the relationship between SERCA activity and whole heart mechanical function is highly non-linear and also involves significant changes in intracellular Ca^2+^ flux (Elliott et al., 2012). Furthermore, it should be noted that in HF, the degree of SERCA impairment varies with disease stage and etiology (Sen et al., 2000) and that restoring the SERCA pump function in HF is not always beneficial (Zhang et al., 2010).

### Impact of Sarco-Endoplasmic Reticulum Ca^2+^-ATPase Inhibition on Spatially Discordant Alternans

SERCA inhibition also caused spatially discordant alternans to occur at slower pacing frequencies (Figure 5). We propose that there is likely a continuum of mechanisms responsible for the onset and progression of alternans. RyR refractoriness is first encroached upon (which as shown in the present study can be indirectly modulated by SERCA activity). This leads to the emergence of SR Ca^2+^ release alternans. As heart rate increases, diastolic SR Ca^2+^ load also begins to alternate, which further augments SR Ca^2+^ release alternans. APD alternans also begins to emerge in this regime. At even faster heart rates, dynamical mechanisms, such as APD and CV restitution, may contribute to spatial discordance and subsequent ventricular tachycardia/fibrillation (VT/VF). The results of this study suggest that simply changing SR Ca^2+^ regulation can shift this entire continuum of mechanisms, in this case, to occur at slower pacing frequencies. Consistent with previous studies, we posit that dynamical mechanisms (i.e., APD or CV restitution) likely still govern the onset of spatial discordance (Wilson and Rosenbaum, 2007). Although CPA did not slow CV, it did prolong APD (due to feedback between SR Ca^2+^ and V_m_, Figure 1). Prolongation of APD will result in shorter diastolic intervals and APD restitution may therefore be invoked at slower pacing frequencies, thereby promoting spatial discordance.

### Sarcoplasmic Reticulum Ca^2+^/V_m_ Coupling

SR Ca^2+^ dynamics can impact APD, as shown in Figure 1, where CPA dose-dependently prolonged APD. Indeed, reduced SR Ca^2+^ release can slow Ca^2+^-dependent inactivation (CDI) of I_CaL_, which would tend to prolong APD. However, a smaller Ca^2+^ transient also tends to reduce the magnitude of inward Na^+^–Ca^2+^ exchange current (I_NCX_), which would tend to shorten APD. Therefore, the net change in APD is a balance between these two mechanisms. The fact that CPA prolongs APD suggests that the net effect of reduced SERCA function (vs. normal SERCA function) on APD is via reduced CDI of I_CaL_. This may seem somewhat at odds with the positive SR Ca^2+^/V_m_ coupling observed during alternans (large SR Ca^2+^ transient corresponds to long APD, Figures 2, 5). However, in the case of beat-to-beat changes in the Ca^2+^ transient, intrinsic SERCA function is the same during large and small beats, and in this case, inward I_NCX_ depends directly on [Ca]_i_ and predominates over I_Ca_ inactivation on APD.

## Conclusion

These findings shed new light on the role of SR Ca^2+^ in the progression from normal rhythms to arrhythmogenic cardiac alternans. While SERCA inhibition caused SR Ca^2+^ and subsequent APD alternans to appear at longer PCLs, this was not directly due to altered diastolic [Ca^2+^]_SR_. However, the reduced SERCA function and consequent lower SR Ca^2+^ load prolonged RyR refractoriness, which is how alternans is promoted by SERCA inhibition. Indeed, SR Ca^2+^ release alternans occurred prior to SR Ca^2+^ load alternans under all conditions, and that can lead secondarily to load alternans, APD alternans, and ultimately spatial discordance. These findings may provide important insight into underlying mechanisms governing alternans onset and severity in failing hearts, where reduced SERCA function is a common phenotype.

## Limitations

This study assessed mechanisms of SR Ca^2+^ and APD alternans but did not specifically address susceptibility to VT/VF nor how SERCA inhibition with CPA may alter VT/VF dynamics. This remains an important area for future study. Although we did not observe appreciable changes in diastolic SR Ca^2+^ load prior to the onset of SR Ca^2+^ release alternans, it is possible that very small changes in diastolic SR Ca^2+^ occur at the luminal side of RyR and govern release. Our imaging approach does not have the spatial resolution nor sensitivity to definitively rule this out.

## Data Availability Statement

The raw data supporting the conclusions of this article will be made available by the authors, without undue reservation.

## Ethics Statement

The animal study was reviewed and approved by the UC Davis Institutional Animal Care and Use Committee.

## Author Contributions

LW and CR conceived the study, designed and conducted experiments, analyzed data, generated figures, and wrote the manuscript. IL analyzed, reviewed and interpreted data, and edited the manuscript. RM and DB contributed to study conception and design, critically reviewed and interpreted data, and edited the manuscript. All authors have reviewed the data and approved the final manuscript.

## Conflict of Interest

The authors declare that the research was conducted in the absence of any commercial or financial relationships that could be construed as a potential conflict of interest.

## Abbreviations

AP: action potential
APD: action potential duration
APD_80_: action potential duration at 80% repolarization
Ca^2+^_i_: intracellular Ca^2+^
[Ca^2+^]_SR_: intra-sarcoplasmic reticulum free Ca^2+^
CICR: Ca^2+^-induced Ca^2+^ release
CPA: cyclopiazonic acid
CV: conduction velocity
ECC: excitation–contraction coupling
ECG: electrocardiogram
I_Ca_: L-type Ca^2+^ current
LV: left ventricle
NCX: Na^+^–Ca^2+^ exchanger
PCL: pacing cycle length
RyR: ryanodine receptor
S1: cycle length of pacing drive train
S2: cycle length of premature pacing stimulus
SERCA: sarco-endoplasmic reticulum Ca^2+^-ATPase
SR: sarcoplasmic reticulum
V_m_: transmembrane potential
VT/VF: ventricular tachycardia/fibrillation.
